# Supplementary material for: JAK-STAT-dependent contact between follicle cells and the oocyte controls Drosophila anterior-posterior polarity and germline development
Source: Nat Commun. 2024 Feb 22;15:1627. doi: 10.1038/s41467-024-45963-z (PMC10883949; doi:10.1038/s41467-024-45963-z)
Supplement: Supplementary file 1 — Supplementary Information [file 41467_2024_45963_MOESM1_ESM.pdf]

Supplementary data

JAK-STAT-dependent contact between follicle cells and the oocyte controls *Drosophila* anterior-posterior polarity and germline development

Charlotte Mallart[1], Sophie Netter[2†], Fabienne Chalvet[1†], Sandra Claret[3], Antoine Guichet[3], Jacques Montagne[1], Anne-Marie Pret[2] and Marianne Malartre[1*]

1 Institute for Integrative Biology of the Cell (I2BC), CEA, CNRS, Université Paris-Saclay, Gif-sur-Yvette, France

2 Institute for Integrative Biology of the Cell (I2BC), CEA, CNRS, Université de Versailles-Saint-Quentin en Yvelines, Université Paris-Saclay, Gif- sur-Yvette, France

3 Université Paris Cité, CNRS, Institut Jacques Monod, Paris, France

[†] These authors have contributed equally to this work

* Correspondence: marianne.malartre@universite-paris-saclay.fr

Content

Supplementary methods 4
1. Antibodies and reagents 4
2. Statistics & reproducibility 5
Supplementary table 1: statistics and reproducibility for Figure 1A-B 5
Supplementary table 2: statistics and reproducibility for Figure 1D,E 5
Supplementary table 3: statistics and reproducibility for Figure 1F,G 5
Supplementary table 4: statistics and reproducibility for Figure 1H-I. 6
Supplementary table 5: reproducibility for Figure 1J. 6
Supplementary table 6: statistics and reproducibility for Figure 1K-L. 6
Supplementary table 7: reproducibility for Figure 2. 7
Supplementary table 8: reproducibility for Figure 3A. 7
Supplementary table 9: statistics and reproducibility for Figure 3B’. 7
Supplementary table 10: statistics and reproducibility for Figure 3C. 8
Supplementary table 11: statistics and reproducibility for Figure 3A’,C’. 8
Supplementary table 12: statistics and reproducibility for Figure 3A’’,D. 9
Supplementary table 13: reproducibility for Figure 3E. 9
Supplementary table 14: statistics and reproducibility for Figure 4A-C. 9
Supplementary table 15: reproducibility for Figure 4D-F. 10
Supplementary table 16: statistics and reproducibility for Figure 5A,A’,D. 10
Supplementary table 17: statistics and reproducibility for Figure 5B. 10
Supplementary table 18: statistics and reproducibility for Figure 5C. 10
Supplementary table 19: statistics and reproducibility for Figure 5E-F. 11
Supplementary table 20: statistics and reproducibility for Figure 5G-H. 11
Supplementary table 21: reproducibility for Figure 6. 11
Supplementary table 22: statistics and reproducibility for Figure 7A-C. 12
Supplementary table 23: statistics and reproducibility for Figure 7A’’,D. 12
Supplementary table 24: reproducibility for Figure 7E. 12
Supplementary table 25: statistics and reproducibility for Figure 7F-G. 12
Supplementary table 26: statistics and reproducibility for Figure 8A-B. 13
Supplementary table 27: statistics and reproducibility for Figure 8A,A’,C. 13
Supplementary table 28: reproducibility for Figure 8D-E’’. 14
Supplementary table 29: statistics and reproducibility for Figure 8F-G. 14
Supplementary table 30: statistics and reproducibility for Supplementary Figure 2. 14
Supplementary table 31: reproducibility for Supplementary Figure 3A-C. 15
Supplementary table 32: statistics and reproducibility for Supplementary Figure 3D-H. 15
Supplementary table 33: reproducibility for Supplementary Figure 4. 15

Supplementary table 34: reproducibility for Supplementary Figure 5A-B. 15
Supplementary table 35: reproducibility for Supplementary Figure 5C,D,F. 16
Supplementary table 36: reproducibility for Supplementary Figure 5E. 16
Supplementary table 37: statistics and reproducibility for Supplementary Figure 5G. 16
Supplementary table 38: reproducibility for Supplementary Figure 6A-B. 17
Supplementary table 39: reproducibility for Supplementary Figure 6C-D. 17
Supplementary table 40: reproducibility for Supplementary Figure 6E. 17
Supplementary table 41: statistics and reproducibility for Supplementary Figure 7A-B. .17
Supplementary table 42: reproducibility for Supplementary Figure 7A,A',C. 18
Supplementary table 43: statistics and reproducibility for Supplementary Figure 7A,A',D. 18
Supplementary table 44: statistics and reproducibility for Supplementary Figure 8. 18
Supplementary table 45: statistics and reproducibility for Supplementary Figure 9. 19

Supplementary figures 20

Supplementary Figure 1. Temporal activity of the *E4-Gal4* driver in PFCs....................21
Supplementary Figure 2. The JAK-STAT pathway is sufficient to define the size of Oskar localization in the oocyte. 22
Supplementary Figure 3. Temporal dynamics of *pnt-LacZ* and *shg-LacZ* expression in the follicular epithelium. 23
Supplementary Figure 4. *oskar* mRNA is restricted to a zone of robust size in the oocyte between stages 9 and 10. 24
Supplementary Figure 5. The JAK-STAT pathway is necessary for *oskar* mRNA anchoring until the end of oogenesis. 25
Supplementary Figure 6. MRLC-2P at the oocyte cortex and high Enabled levels in PFC restrict between stages 7 and 10. 26
Supplementary Figure 7. E-Cadherin acts downstream of JAK-STAT signaling to maintain PAC-oocyte membranes tight for *oskar* mRNA anchoring in the oocyte. 27
Supplementary Figure 8. *shotgun* is sufficient for ectopic JAK-STAT signaling in PFCs and acts in parallel to *enabled* in PAC differentiation. 28
Supplementary Figure 9. *enabled* is sufficient to maintain MRLC-2P at the oocyte posterior cortex..........................29

Supplementary methods

1. Antibodies and reagents

Primary antibodies					
**Target**	**Host species**	**Dilution**	**Company name**	**Catalog number**	**Validation**
GFP	Rabbit	1:200	Interchim	FP-37151B	Tested on transfected cells by manufacturer
GFP	Mouse	1:50	DSHB	DSHB-GFP-1D2	Tested on recombinant GFP expressed in E. coli by manufacturer
Staufen	Goat	1:200	Santa Cruz	sc-15823	Tested on Schneider' Drosophila Line 2 wholecell lysate by manufacturer
E-Cadherin	Rat	1:200	DSHB	DCAD2	Original publication DOI: 10.1006/dbio.1994.1287.
Beta-galactosidase	Mouse	1:200	DSHB	40-1a	DOI: 10.1128/mcb.11.12.5848-5859.1991
Vasa	Rat	1:200	DSHB	anti-vasa	Original publication DOI: 10.1534/genetics.108.100057
Enabled	Mouse	1:150	DSHB	5G2 anti-enabled	Original publication DOI:10.1016 /s0092-8674(00)80883-1

Target	Host species	Dilution	Original publication	Gift from
Oskar	Rabbit	1:1000	DOI:10.1016/0092-8674(91)90137-n	A. Ephrussi
MRLC-2P	Rat	1:500	DOI:10.1016/j.gep.2010.09.008	R.E. Ward

Secondary antibodies					
**Target**	**Dye**	**Dilution**	**Company name**	**Catalog number**	**Validation**
Mouse	Cy3	1:200	Jackson Laboratories	115-165-003	Tested by immunoelectrophoresis and/or ELISA by manufacturer
Rat	Alexa 647	1:200	Jackson Laboratories	112-605-167	Tested by immunoelectrophoresis and/or ELISA by manufacturer
Rabbit	Alexa 488	1:200	Thermo Fisher Scientific	A-11008	Tested by immunohistochemistry and immunocytochemistry by manufacturer
Goat	Alexa 488	1:200	Thermo Fisher Scientific	A-11055	Tested by flow cytometry, immunohistochemistry and immunocytochemistry by manufacturer
Goat	Alexa 568	1:200	Thermo Fisher Scientific	A-11057	Tested by flow cytometry, immunohistochemistry and immunocytochemistry by manufacturer
Rat	Alexa 647	1:200	Thermo Fisher Scientific	A-21247	Tested by western blot, immunocytochemistry and immunoprecipitation by manufacturer

Reagents				
**Name**	**Dilution**	**Company name**	**Catalog number**	**Purpose**
Phalloidin–Atto 647N	1:200	Sigma-Aldrich	65906	Actin labeling
DAPI	1ng/ml final concentration			DNA labeling
DABCO	5% with 70% glycerol	Sigma-Aldrich	D27802	Mounting medium

2. Statistics & reproducibility

Sample size (n) corresponds to the total number of cases analyzed, grouped from all independent experiments.

Supplementary table 1: statistics and reproducibility for Figure 1A-B.

Genotypes	Nb of independent experiments	Nb of embryos analyzed (n)
E4>	3	22
E4>hop^{TUM}	5	39

Statistical test	Compared conditions	P value	Attributed stars
two-sided Fisher's exact test	0-40 PGCs	0.001	***
	>40 PGCs		

Supplementary table 2: statistics and reproducibility for Figure 1D,E.

Genotype	Stages	Nb of independent experiments	Nb of follicles analyzed (n)
fru>	9	2	13
	10a		17
	Early 10b		17
	Late 10b		14

Statistical test	Compared conditions	P value	Attributed stars
two-sided Mann-Whitney	St9	0.0074	**
	St10a		
	St10a	0.4327	ns
	Early St10b		
	Early St10b	0.9999	ns
	Late St10b		

Supplementary table 3: statistics and reproducibility for Figure 1F,G.

Genotypes	Stages	Nb of independent experiments	Nb of follicles analyzed (n)
E4>	10a	3	14
	Early 10b		16
	Late 10b		14
E4>hop^{TUM}	10a	2	14
	Early 10b		7
	Late 10b		5

Measurements	Statistical tests	Compared conditions		P value	Attributed stars
# of FCs around PCs	two-sided Mann-Whitney	St10a	*E4>*	0.0007	***
			E4>hop^{TUM}		
		Early St10b	*E4>*	0.0007	***
			E4>hop^{TUM}		
		Late St10b	*E4>*	0.006	**
			E4>hop^{TUM}		
		E4>hop^{TUM}	St10a	0.8064	ns
			Early St10b		
			Early St10b	0.2487	ns
			Late St10b		
Diameter	two-sided unpaired t-test	St10a	*E4>*	<0.0001	****
			E4>hop^{TUM}		
		Early St10b	*E4>*	<0.0001	****
			E4>hop^{TUM}		
		Late St10b	*E4>*	<0.0001	****
			E4>hop^{TUM}		

Supplementary table 4: statistics and reproducibility for Figure 1H-I.

Genotype	Stages	Nb of independent experiments	Nb of follicles analyzed (n)
10XSTAT92E-GFP	Late 8	1	22
	9		21
	10		7

Statistical test	Compared conditions	P value	Attributed stars
two-sided unpaired t-test	Late St8	0.0021	**
	St9		
	St9	0.9311	ns
	St10		

Supplementary table 5: reproducibility for Figure 1J.

Genotype	Nb of independent experiments	Nb of follicles analyzed (n)
fru>	2	27

Supplementary table 6: statistics and reproducibility for Figure 1K-L.

Genotypes	Nb of independent experiments	Nb of follicles analyzed (n)
10XSTAT92E-GFP;E4>	1	18
10XSTAT92E-GFP;E4>hop^{TUM}		11

Statistical test	Compared conditions	P value	Attributed stars
two-sided unpaired t-test	*10XSTAT92E-GFP;E4>*	<0.0001	****
	10XSTAT92E-GFP;E4>hop^{TUM}		

Supplementary table 7: reproducibility for Figure 2.

Panel	Genotypes/conditions	Stage	Nb of independent experiments		Nb of follicles analyzed (n)
A	*shg-LacZ* and *shg-LacZ/+*	8	3		19
B	*nos>shg-RNAi*	8-9	4		33
C	*grk-*	8-10a	2		29
D	EGFR-RNAi clones	8-10a	RNAi line #JF01084	2	57
			RNAi line #KK100051	2	
E	*shg-LacZ*	10	1		9
E'	*shg-LacZ/+;upd>upd-RNAi*				16
F	*E4>*	10	3		39
F'	*E4>hop^{TUM}*				35

Supplementary table 8: reproducibility for Figure 3A.

Panel	Genotype	Stages	Nb of independent experiments		Nb of follicles analyzed (n)
A	*w1118*	10a	Staufen anchoring	1	5
			Perivitelline space	2	22

Supplementary table 9: statistics and reproducibility for Figure 3B'.

Panel	Genotype	Stages	Nb of independent experiments	Nb of mean space measurements on each side of PCs (n)
B'	*fru>*	9	4	24
		10a		54
		Early 10b		50
		Late 10b		16

Statistical test	Compared conditions		P value	Attributed stars
two-sided unpaired t-test	Late St9	Zone 1	0.0002	***
		Zone 2		
	St10a	Zone 1	<0.0001	****
		Zone 2		
	Early St10b	Zone 1	<0.0001	****
		Zone 2		
	Late St10b	Zone 1	0.0065	**
		Zone 2		

Supplementary table 10: statistics and reproducibility for Figure 3C.

Genotypes	Nb of independent experiments	Nb of mean space measurements on each side of PCs (n)
upd-Gal4/+ and *upd-Gal4*/+;*shg-LacZ/+*	4	22
upd-Gal4/+;*upd-RNAi/shg-LacZ* and *upd-Gal4*/+;*upd-RNAi/+*		22

Statistical test	Compared conditions		P value	Attributed stars
two-sided unpaired t-test	PACs	Control	<0.000001	****
		upd>upd-RNAi		
	Zone 2	Control	<0.000001	****
		upd>upd-RNAi		
	Zone 3	Control	<0.000001	****
		upd>upd-RNAi		
	Zone 4	Control	0.00143	**
		upd>upd-RNAi		
	Zone 5	Control	0.006474	**
		upd>upd-RNAi		

Supplementary table 11: statistics and reproducibility for Figure 3A’,C’.

For reproducibility of Staufen anchoring phenotype presented in A’, see Supplementary table 33.

Genotypes	Nb of independent experiments	Nb of mean space measurements on each side of PCs (n)
upd-Gal4/+ and *upd-Gal4*/+;*shg-LacZ/+*	4	45
upd-Gal4/+;*upd-RNAi/shg-LacZ* and *upd-Gal4*/+;*upd-RNAi/+*		41

Statistical test	Compared conditions		P value	Attributed stars
two-sided unpaired t-test	PACs	Control	<0.000001	****
		upd>upd-RNAi		
	Zone 2	Control	<0.000001	****
		upd>upd-RNAi		
	Zone 3	Control	<0.000001	****
		upd>upd-RNAi		
	Zone 4	Control	0.000216	***
		upd>upd-RNAi		
	Zone 5	Control	0.028032	*
		upd>upd-RNAi		

Supplementary table 12: statistics and reproducibility for Figure 3A'',D.

Genotypes	Nb of independent experiments	Nb of mean space measurements on each side of PCs (n)
E4>	4	46
E4>hop^{TUM}		38

Statistical test	Compared conditions		P value	Attributed stars
two-sided unpaired t-test	PACs	*E4>*	0.000081	****
		E4>hop^{TUM}		
	Zone 2	*E4>*	<0.000001	****
		E4>hop^{TUM}		
	Zone 3	*E4>*	<0.000001	****
		E4>hop^{TUM}		
	Zone 4	*E4>*	<0.000001	****
		E4>hop^{TUM}		
	Zone 5	*E4>*	0.000013	****
		E4>hop^{TUM}		

Supplementary table 13: reproducibility for Figure 3E.

Genotypes	Nb of independent experiments	Nb of follicles analyzed (n)
10XSTAT92E-GFP;E4>hop^{TUM}	1	11

Supplementary table 14: statistics and reproducibility for Figure 4A-C.

Genotypes	Nb of independent experiments	Nb of follicles analyzed (n)
w1118	2	14
upd>upd-RNAi		4
E4>	2	11
E4>hop^{TUM}		8

Statistical test	Compared conditions	P value	Attributed stars
two-sided Mann-Whitney	*w1118*	0.0003	***
	upd>upd-RNAi		
	E4>	<0.0001	****
	E4>hop^{TUM}		

Supplementary table 15: reproducibility for Figure 4D-F.

Genotypes	Nb of independent experiments	Stages	Nb of follicles analyzed (n)
Khc::LacZ;upd>	2	9	11
		10a	6
*Khc::LacZ;upd>*upd-RNAi		9	11
		10a	6

Supplementary table 16: statistics and reproducibility for Figure 5A,A',D.

Genotypes	Stages	Nb of independent experiments	Nb of follicles analyzed (n)
E4>	10a	3	14
	Early 10b		16
	Late 10b		14
E4>shg	10a	2	22
	Early 10b		15
	Late 10b		11

Statistical test	Compared conditions		P value	Attributed stars
two-sided Mann-Whitney	St10a	*E4>*	<0.0001	****
		E4>shg		
	Early St10b	*E4>*	<0.0001	****
		E4>shg		
	Late St10b	*E4>*	<0.0001	****
		E4>shg		
	E4>shg	St10a	0.2782	ns
		Early St10b		
		Early St10b	0.3215	ns
		Late St10b		

Supplementary table 17: statistics and reproducibility for Figure 5B.

Genotypes	Nb of independent experiments	Nb of follicles analyzed (n)
shg clones	2	22

Supplementary table 18: statistics and reproducibility for Figure 5C.

Genotypes	Nb of independent experiments	Nb of mean space measurements on each side of PCs (n)
E4>	4	46
E4>shg		60

Statistical test	Compared conditions		P value	Attributed stars
two-sided unpaired t-test	PACs	*E4>*	0.020801	*
		E4>shg		
	Zone 2	*E4>*	0.000064	****
		E4>shg		
	Zone 3	*E4>*	0.0059	**
		E4>shg		
	Zone 4	*E4>*	0.000015	****
		E4>shg		
	Zone 5	*E4>*	0.034197	*
		E4>shg		

Supplementary table 19: statistics and reproducibility for Figure 5E-F.

Genotypes	Nb of independent experiments	Nb of follicles analyzed (n)
E4>	2	11
E4>shg		14

Statistical test	Compared conditions	P value	Attributed stars
two-sided Mann-Whitney	*E4>*	<0.0001	****
	E4>shg		

Supplementary table 20: statistics and reproducibility for Figure 5G-H.

Genotypes	Nb of independent experiments	Nb of embryos analyzed (n)
E4>	1	88
E4>shg		60

Statistical test	Compared conditions	P value	Attributed stars
two-sided Fisher's exact test	0-40 PGCs	<0.0001	****
	>40 PGCs		

Supplementary table 21: reproducibility for Figure 6.

Panel	Nb of independent experiments	Nb of follicles analyzed (n)
A	2	5
B-C	1	2
D-E	1	1

Supplementary table 22: statistics and reproducibility for Figure 7A-C.

Genotypes	Nb of independent experiments	Nb of follicles analyzed (n)
w1118	2	7
upd>upd-RNAi		5
E4>	2	8
E4>hop^{TUM}		10

Statistical test	Compared conditions	P value	Attributed stars
two-sided Mann-Whitney	*w1118*	0.0013	**
	upd>upd-RNAi		
	E4>	<0.0001	****
	E4>hop^{TUM}		

Supplementary table 23: statistics and reproducibility for Figure 7A'',D.

Genotypes	Nb of independent experiments	Number of zones on each side of PCs analyzed
E4>	1	6
E4>hop^{TUM}		8

Statistical test	Compared conditions		P value	Attributed stars
two-sided Fisher's exact test	PACs	*E4>*	0.9999	ns
		E4>hop^{TUM}		
	Zone 2	*E4>*	0.0005	***
		E4>hop^{TUM}		
	Zone 3	*E4>*	<0.0001	****
		E4>hop^{TUM}		

Supplementary table 24: reproducibility for Figure 7E.

Genotypes	Nb of independent experiments	Nb of follicles analyzed (n)
hop^{TUM} clones	1	7

Supplementary table 25: statistics and reproducibility for Figure 7F-G.

Genotypes	Nb of independent experiments	Number of zones on each side of PCs analyzed
upd>;E4>mCD8::GFP	1	5
upd>;E4>mCD8::GFP;upd-RNAi		9

Statistical test	Compared conditions		P value	Attributed stars
two-sided Fisher's exact test	PACs	*upd>;E4>mCD8::GFP*	0.0002	***
		upd>;E4>mCD8::GFP;upd-RNAi		

Supplementary table 26: statistics and reproducibility for Figure 8A-B.

Genotypes	Stages	Nb of independent experiments	Nb of follicles analyzed (n)
E4>	10a	3	23
	Early 10b		19
E4>ena	10a		22
	Early 10b		21

Statistical test	Compared conditions		P value	Attributed stars
two-sided Mann-Whitney	St10a	*E4>*	<0.0001	****
		E4>ena		
	Early St10b	*E4>*	0.0388	*
		E4>ena		
	E4>ena	St10a	0.4263	ns
		Early St10b		

Supplementary table 27: statistics and reproducibility for Figure 8A,A',C.

Genotypes	Nb of independent experiments	Nb of mean space measurements on each side of PCs (n)
E4>	3	42
E4>ena		34

Statistical test	Compared conditions		P value	Attributed stars
two-sided unpaired t-test	PACs	*E4>*	0.000203	***
		E4>ena		
	Zone 2	*E4>*	0.000068	****
		E4>ena		
	Zone 3	*E4>*	0.001409	**
		E4>ena		
	Zone 4	*E4>*	0.0129	*
		E4>ena		
	Zone 5	*E4>*	0.031457	*
		E4>ena		

Supplementary table 28: reproducibility for Figure 8D-E''.

Genotypes	Nb of independent experiments	Nb of follicles analyzed (n)
E4>	1	8
E4>ena		16
Ena GOF clones	1	21

Supplementary table 29: statistics and reproducibility for Figure 8F-G.

Genotypes	Nb of independent experiments	Nb of embryos analyzed (n)
E4>	1	88
E4>ena		97

Statistical test	Compared conditions	P value	Attributed stars
two-sided Fisher's exact test	0-40 PGCs	<0.0001	****
	>40 PGCs		

Supplementary table 30: statistics and reproducibility for Supplementary Figure 2.

Genotypes	Stages	Nb of independent experiments	Nb of follicles analyzed (n)
E4>	10a	4	17
	Early 10b		15
	Late 10b		12
E4>hop^{TUM}	10a	3	15
	Early 10b		8
	Late 10b		11

Measurements	Statistical tests	Compared conditions		P value	Attributed stars
# of FCs around PCs	two-sided Mann-Whitney	St10a	*E4>*	<0.0001	****
			E4>hop^{TUM}		
		Early St10b	*E4>*	<0.0001	****
			E4>hop^{TUM}		
		Late St10b	*E4>*	0.0451	*
			E4>hop^{TUM}		
		E4>hop^{TUM}	St10a	0.5919	ns
			Early St10b		
			Early St10b	0.0448	*
			Late St10b		
Diameter	two-sided unpaired t-test	St10a	*E4>*	0.0089	**
			E4>hop^{TUM}		
		Early St10b	*E4>*	0.0071	**
			E4>hop^{TUM}		
		Late St10b	*E4>*	0.9509	ns
			E4>hop^{TUM}		

Supplementary table 31: reproducibility for Supplementary Figure 3A-C.

Stages	Nb of independent experiments	Nb of follicles analyzed (n)
7	1	12
10		7

Supplementary table 32: statistics and reproducibility for Supplementary Figure 3D-H.

Genotypes	Stages	Nb of independent experiments	Nb of follicles analyzed (n)
shg-LacZ (homozygous)	7	1	8
	8		17
	9		10
	10		14
shg-LacZ/+	10	1	9
shg-LacZ/+ ; upd>upd-RNAi			16

Statistical test	Compared conditions	P value	Attributed stars
two-sided Mann-Whitney	*shg-LacZ/+*	<0.0001	****
	shg-LacZ/+ ; upd>upd-RNAi		

Supplementary table 33: reproducibility for Supplementary Figure 4.

Quantifications of Staufen anchoring zone size and perivitelline space are presented in Figure 1E and Figure 3B' respectively.

Genotype	Phenotypes	Stages	Nb of independent experiments	Nb of follicles analyzed (n)
fru>	Staufen anchoring zone	9	2	13
		Early 10b		17
		Late 10b		14
	Perivitelline space			Nb of mean space measurements on each side of PCs (n)
		9	4	24
		Early 10b		54
		Late 10b		50

Supplementary table 34: reproducibility for Supplementary Figure 5A-B.

Genotypes	Nb of independent experiments	Staufen anchoring phenotypes	Nb of follicles analyzed (n)
w1118	4	No defect	11
upd>upd-RNAi		Not anchored	5
		Misplaced	4
		Partial	12
		Small	6
		No defect	16

Supplementary table 35: reproducibility for Supplementary Figure 5C,D,F.

Genotypes	Stages	Nb of independent experiments	Staufen anchoring phenotypes	Nb of follicles analyzed (n)
oskar::GFP	10	2	No defect	25
	14			55
oskar::GFP ; upd>upd-RNAi	10		Not anchored	3
			Misplaced	0
			Partial	3
			Small	10
			No defect	1
	14		Not anchored	7
			Misplaced	14
			Partial	11
			Small	53
			No defect	16

Supplementary table 36: reproducibility for Supplementary Figure 5E.

Genotypes	Stages	Nb of independent experiments	Nb of follicles analyzed (n)
oskar::GFP	10a	2	10
	Early 10b		15

Supplementary table 37: statistics and reproducibility for Supplementary Figure 5G.

Genotypes	Stages	Nb of independent experiments	Nb of follicles analyzed (n)
oskar::GFP	12	2	9
	13		13
	14		55
oskar::GFP ; upd>upd-RNAi	12		9
	13		12
	14		80

Statistical test	Compared conditions		P value	Attributed stars
two-sided unpaired t-test	St12	*oskar::GFP*	0,0121	*
		oskar::GFP ; upd>upd-RNAi		
	St13	*oskar::GFP*	0,0014	**
		oskar::GFP ; upd>upd-RNAi		
	St14	*oskar::GFP*	<0,0001	****
		oskar::GFP ; upd>upd-RNAi		

Supplementary table 38: reproducibility for Supplementary Figure 6A-B.

Genotypes	Stages	Nb of independent experiments	Nb of follicles analyzed (n)
w1118	6-7	2	15
	Early 8		12
	Late 8		13
	Early 9		24
	Late 9		13
	10		21

Supplementary table 39: reproducibility for Supplementary Figure 6C-D.

Genotypes	Stages	Nb of independent experiments	Nb of follicles analyzed (n)
w1118	6-7	2	10
	Early 8		10
	Late 8		17
	Early 9		26
	Late 9		12
	10		13

Supplementary table 40: reproducibility for Supplementary Figure 6E.

Genotypes	Nb of independent experiments	Number of follicles analyzed
tj>mCD8::GFP	1	3

Supplementary table 41: statistics and reproducibility for Supplementary Figure 7A-B.

Genotypes	Stages	Nb of independent experiments	Nb of follicles analyzed (n)
upd;E4>LacZ	10a	3	9
upd;E4>upd-RNAi;LacZ			20
upd;E4>upd-RNAi;shg			10
upd;E4>LacZ;shg			20

Statistical test	Compared conditions	P value	Attributed stars
two-sided Mann-Whitney	*upd;E4>LacZ*	0.0058	**
	upd;E4>upd-RNAi;LacZ		
	upd;E4>upd-RNAi;LacZ	0.0353	*
	upd;E4>upd-RNAi;shg		
	upd;E4>LacZ	0.6904	ns
	upd;E4>upd-RNAi;shg		

Supplementary table 42: reproducibility for Supplementary Figure 7A,A’,C.

Genotypes	Nb of independent experiments	Staufen anchoring phenotypes	Nb of follicles analyzed (n)
upd;E4>upd-RNAi;LacZ	3	Not anchored	9
		Misplaced	1
		Partial	5
		Small	14
		No defect	14
upd;E4>upd-RNAi;shg		Not anchored	1
		Misplaced	0
		Partial	0
		Small	5
		No defect	11

Supplementary table 43: statistics and reproducibility for Supplementary Figure 7A,A’,D.

Genotypes	Nb of independent experiments	Nb of mean space measurements on each side of PCs (n)
upd;E4>LacZ	3	16
upd;E4>upd-RNAi;LacZ		24
upd;E4>upd-RNAi;shg		18
upd;E4>LacZ;shg		38

Statistical test	Compared conditions		P value	Attributed stars
two-sided unpaired t-test	PACs	*upd;E4>upd-RNAi;LacZ*	0.007129	**
		upd;E4>upd-RNAi;shg		
	Zone 2	*upd;E4>upd-RNAi;LacZ*	0.004015	**
		upd;E4>upd-RNAi;shg		
	Zone 3	*upd;E4>upd-RNAi;LacZ*	0.081449	ns
		upd;E4>upd-RNAi;shg		
	Zone 4	*upd;E4>upd-RNAi;LacZ*	0.402903	ns
		upd;E4>upd-RNAi;shg		
	Zone 5	*upd;E4>upd-RNAi;LacZ*	0.084207	ns
		upd;E4>upd-RNAi;shg		

Supplementary table 44: statistics and reproducibility for Supplementary Figure 8.

Panels	Genotypes	Nb of independent experiments	Nb of follicles analyzed (n)
A,B	*E4>*	1	8
A’,B	*E4>ena*		16
C,D	*E4>*	2	9
C’,D	*E4>shg*		15
E,F	*10XSTAT92E-GFP;E4>*	2*	21**
E’,F	*10XSTAT92E-GFP;E4>shg*		21**

* Only one experiment plotted in the graph presented in (F). Similar results were obtained in the second experiment.

** n for the experiment plotted in (F).

Statistical tests	Panels	Compared conditions	P value	Attributed stars
two-sided Mann-Whitney	B	*E4>*	0.0654	ns
		E4>ena		
	D	*E4>*	<0.0001	****
		E4>shg		
two-sided unpaired t-test	F	*10XSTAT92E-GFP;E4>*	<0.0001	****
		10XSTAT92E-GFP;E4>shg		

Supplementary table 45: statistics and reproducibility for Supplementary Figure 9.

Genotypes	Nb of independent experiments	Nb of follicles analyzed (n)
E4>	2	9
E4>ena		15

Statistical test	Compared conditions	P value	Attributed stars
two-sided Mann-Whitney	*E4>*	0.0132	*
	E4>ena		

Supplementary figures

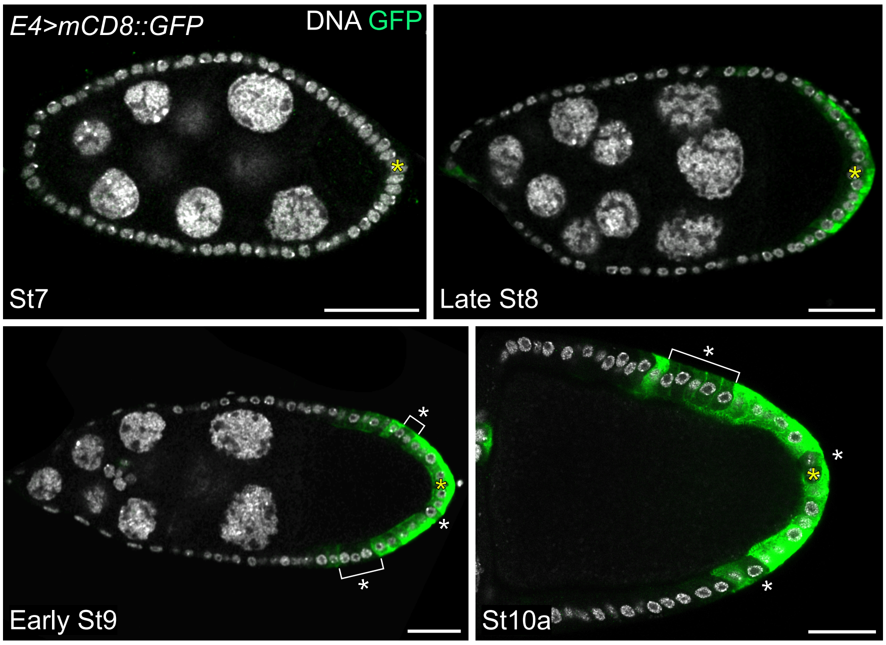

Supplementary Figure 1. Temporal activity of the *E4-Gal4* driver in PFCs. Posterior is to the right. PCs are indicated with yellow stars. Follicles expressing *mCD8-GFP* under the control of *E4-Gal4* showing GFP (green) staining starting at stage 8. DNA is in grey (DAPI). Brackets and white stars highlight FCs with no GFP within the expression domain, reflecting the mosaicism of the driver.
St: stage. Scale: 30µm.

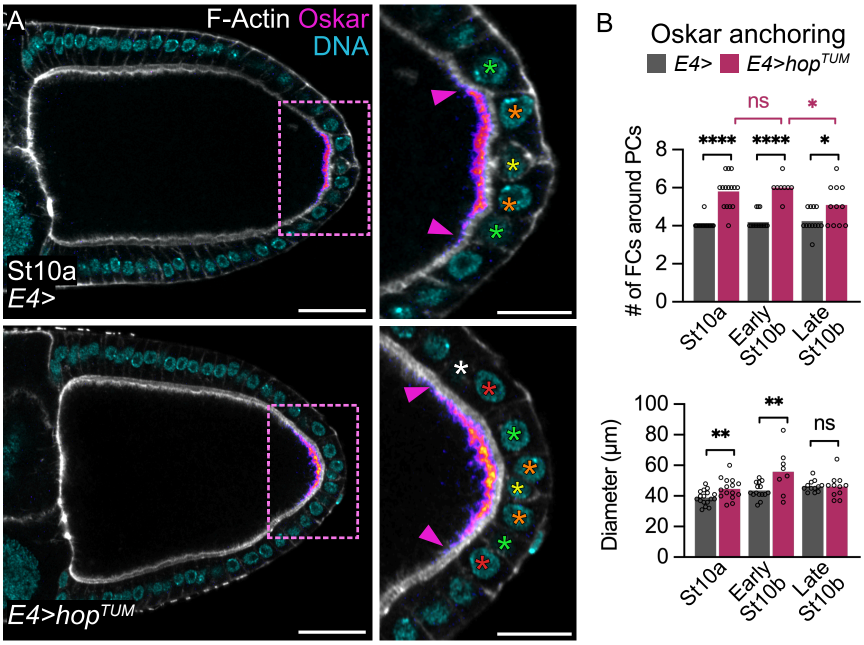

Supplementary Figure 2. The JAK-STAT pathway is sufficient to define the size of Oskar localization in the oocyte. (A) Posterior is to the right. Control follicle (*E4/+*) and follicle expressing *hop*^{TUM} in PFCs under the control of *E4-Gal4* stained for F-Actin (grey, phalloidin), DNA (cyan, DAPI) and Oskar (fire filter). Oskar anchoring zones (pink dotted rectangles) are magnified. Yellow, orange, green, red and white stars indicate PCs, PC+1, +2, +3 and +4 rows respectively. Oskar anchoring zone boundary is indicated by pink arrowheads. **(B)** Quantifications of Oskar anchoring zone as the number of FCs around PCs facing Oskar signal and in diameter in control follicles and follicles expressing *hop*^{TUM} in PFCs.
St: stage. #: number. Scale: 30µm except magnifications 15µm. Statistical tests: two-sided unpaired t-test for Diameter; two-sided Mann-Whitney for # of FCs around PCs. Information about statistics and reproducibility is provided in Supplementary table 30. The data underlying the figure is provided as a Source Data file.

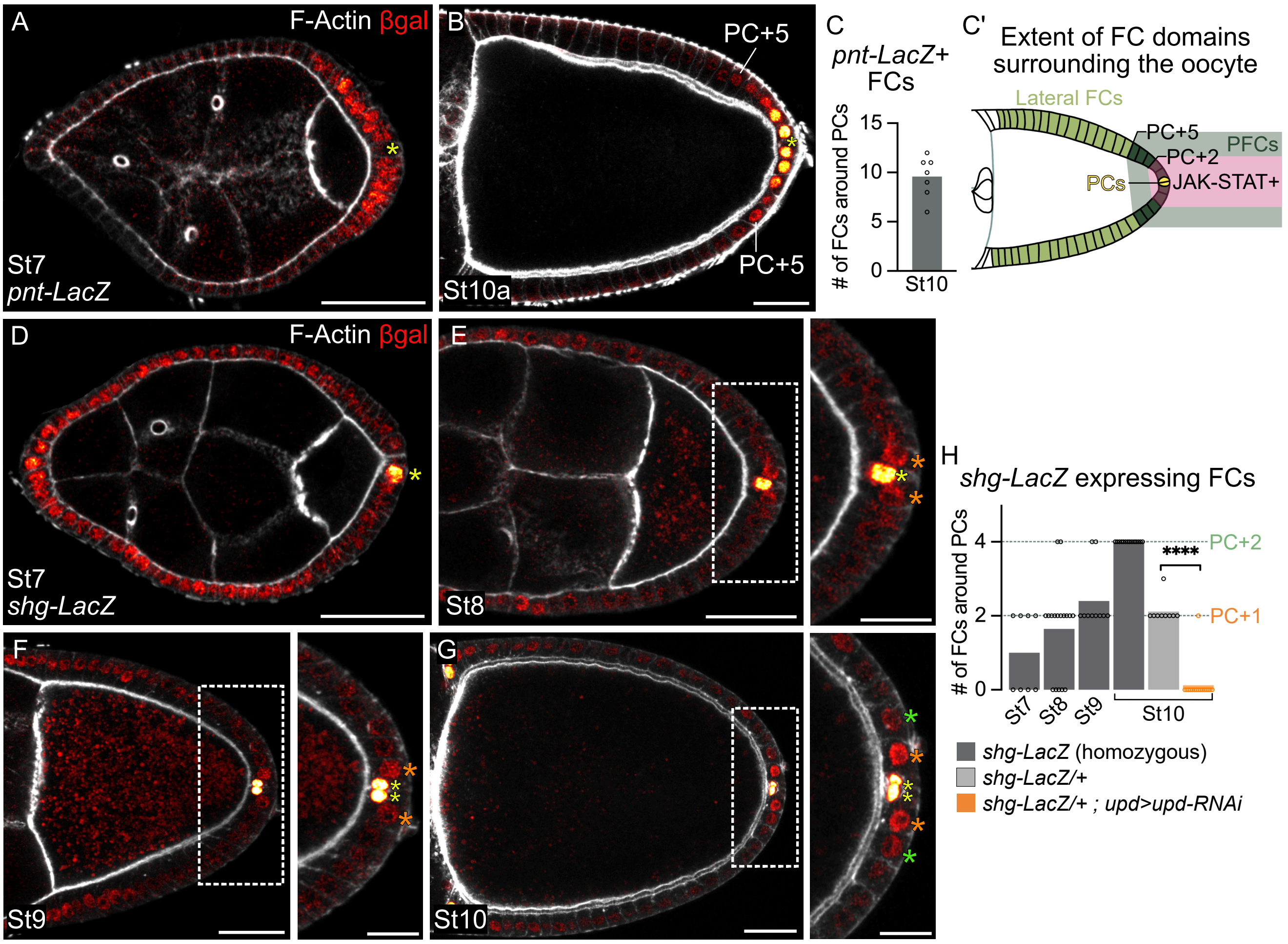

Supplementary Figure 3. Temporal dynamics of *pnt-LacZ* and *shg-LacZ* expression in the follicular epithelium. Posterior is to the right. Yellow stars indicate PCs. Stage 10 are max projections of 3 consecutive 1µm confocal sections passing through PCs. **(A,B)** Follicles expressing *pnt-LacZ* stained for F-Actin (grey, phalloidin) and galactosidase (red hot filter). **(C,C')** Quantification of the number of *pnt-LacZ* expressing FCs around PCs at stage 10, corresponding to the PFC domain that includes a subpopulation of JAK-STAT active cells, as represented in (C'). The other FCs around the oocyte are referred to as lateral FCs. **(D-G)** Follicles expressing the *shg-LacZ* reporter stained for F-Actin (white, phalloidin) and galactosidase (red hot filter). PFCs (white dotted rectangles) are magnified. Orange and green stars indicate PC+1 and +2 rows, respectively, where *shg-LacZ* is expressed at high levels. Pattern of low *shg-LacZ* expression in PFCs is observed in 100% of stage 7 follicles analyzed. **(H)** Quantification of the number of PFCs expressing *shg-LacZ* around PCs in indicated genotypes.
#: number. St: stage. Scale: 30µm except magnifications 15µm. Information about statistics and reproducibility is provided in Supplementary tables 31-32. The data underlying the figure is provided as a Source Data file.

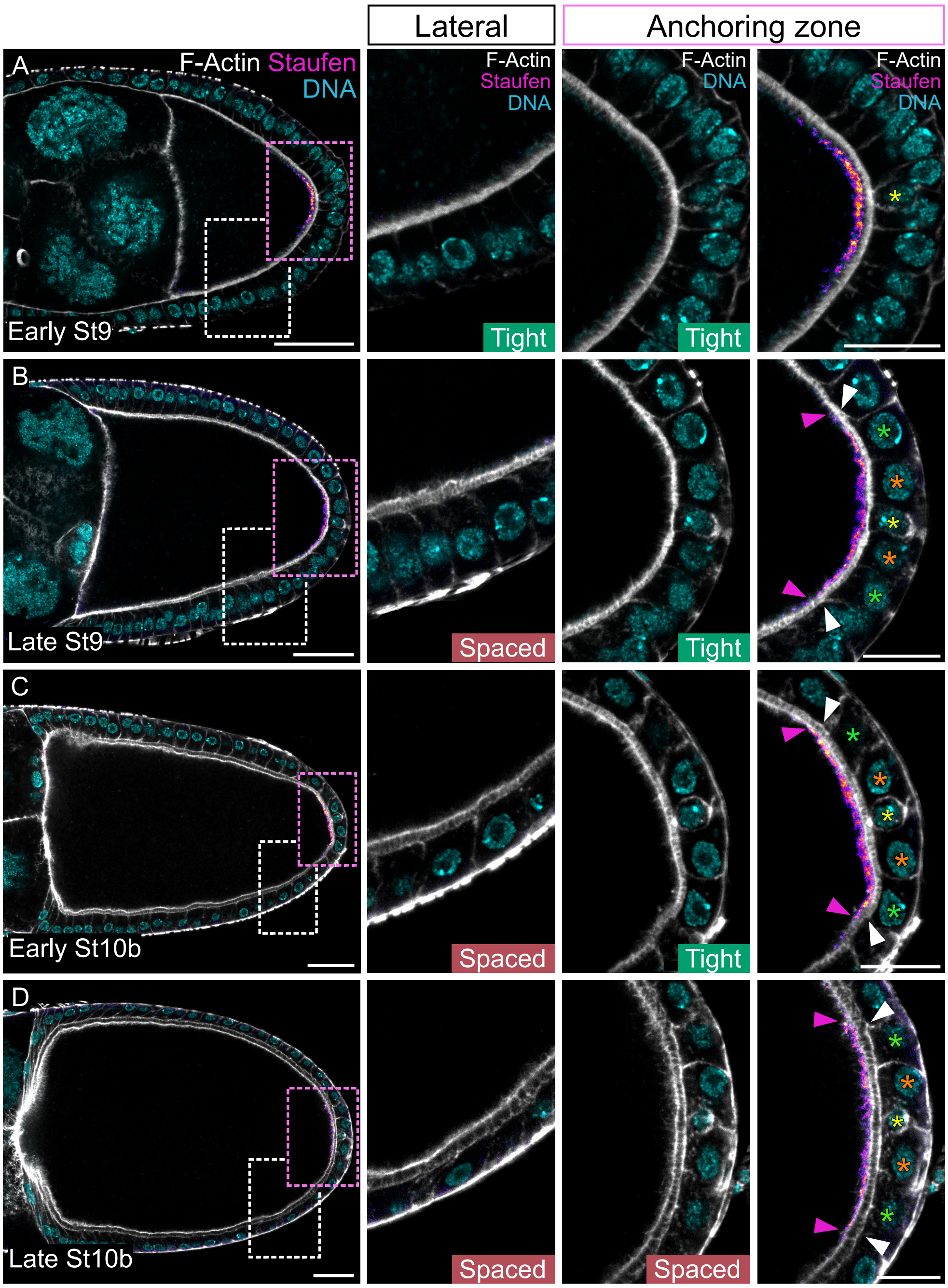

Supplementary Figure 4. *oskar* mRNA is restricted to a zone of robust size in the oocyte between stages 9 and 10. Posterior is to the right. *oskar* mRNA anchoring zone (pink dotted rectangles) and adjacent lower lateral region (white dotted rectangles) are magnified. Yellow, orange and green stars indicate PCs, PC+1 and +2 rows respectively. Arrowheads indicate the anchoring zone boundary on oocyte (pink) and FC (white) sides. “Tight” and “Spaced” refer to the perivitelline space. F-Actin (grey, phalloidin), DNA (cyan, DAPI) and Staufen (fire filter) staining of control follicles (*fruitless-Gal4*/+).
St: stage. Scale: 30µm except magnifications 15µm. Information about reproducibility is provided in Supplementary table 33.

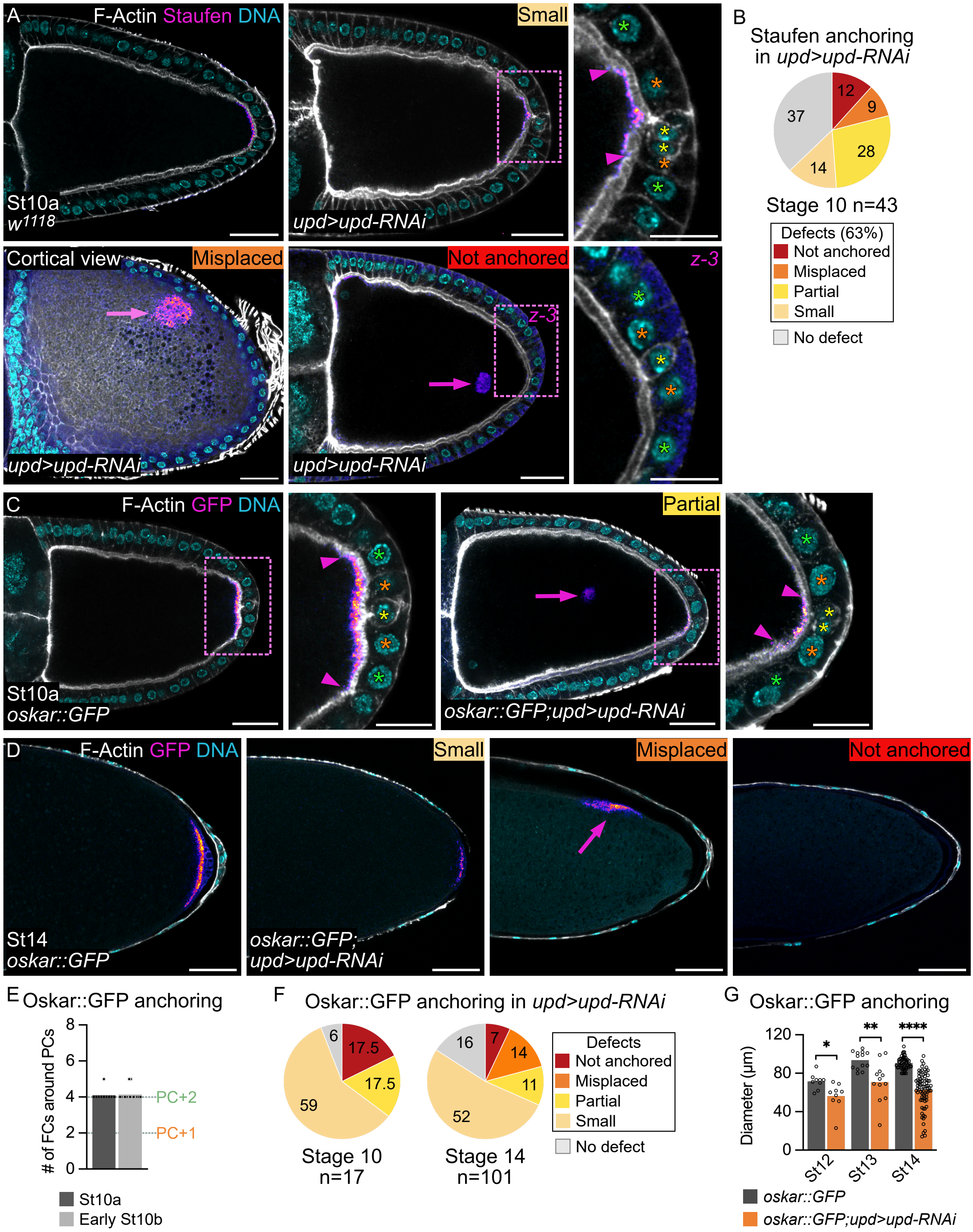

Supplementary Figure 5. The JAK-STAT pathway is necessary for *oskar* mRNA anchoring until the end of oogenesis. Posterior is to the right. Follicles are stained with F-Actin (grey, phalloidin) and DNA (cyan, DAPI). Yellow, orange and green stars indicate PCs and PC+1 and +2 rows respectively. PACs (pink dotted rectangles) are magnified. Pink arrowheads indicate the boundary of Oskar anchoring zone in the oocyte. (A) “Misplaced” and (C) “Partial” are max z-projection of 3 consecutive 1µm confocal sections. Pink arrows point to abnormal Staufen or Oskar localizations. Not anchored: dot in the oocyte cytoplasm. Misplaced: at the oocyte cortex but not in the posterior anchoring zone. Small: anchoring zone size <4 PFCs and <30-40-80 µm at stage 10a-10b-14 respectively. Partial: both small anchoring zone and not anchored phenotypes. **(A)** Staufen (fire filter) staining in control follicles (w^{1118}), and follicles expressing *upd-RNAi* in PCs under the control of *upd-Gal4*. Staufen localization phenotypes quantified in (B) are indicated in color boxes. In “Not anchored”, magnification from *z-3* confocal section passing through PCs shows the widened perivitelline space. **(B)** Diagram presenting Staufen localization phenotype categories observed in *upd>upd-RNAi* stage 10 follicles. Those defects were never observed in control follicles. **(C,D)** Expression of *oskar::GFP* in control follicles and in follicles expressing *upd-RNAi* in PCs. Native GFP in fire filter. Oskar::GFP localization phenotypes are indicated in color boxes and quantified in **(F)**. Those defects were never observed in control follicles. **(E)** Quantifications of the size of Oskar anchoring zone as the number of FCs around PCs facing Oskar::GFP signal. **(G)** Quantification of the size of Oskar::GFP anchoring zone in diameter in control follicles and follicles expressing *upd-RNAi* in PCs presenting “Partial”, “Small” and “No defect” phenotypes.
St: stage. Scale: (A,C) 30µm except magnifications 15µm; (D) 50µm. Statistical tests: two-sided unpaired t-test. Information about statistics and reproducibility is provided in Supplementary tables 34-37. The data underlying the figure is provided as a Source Data file.

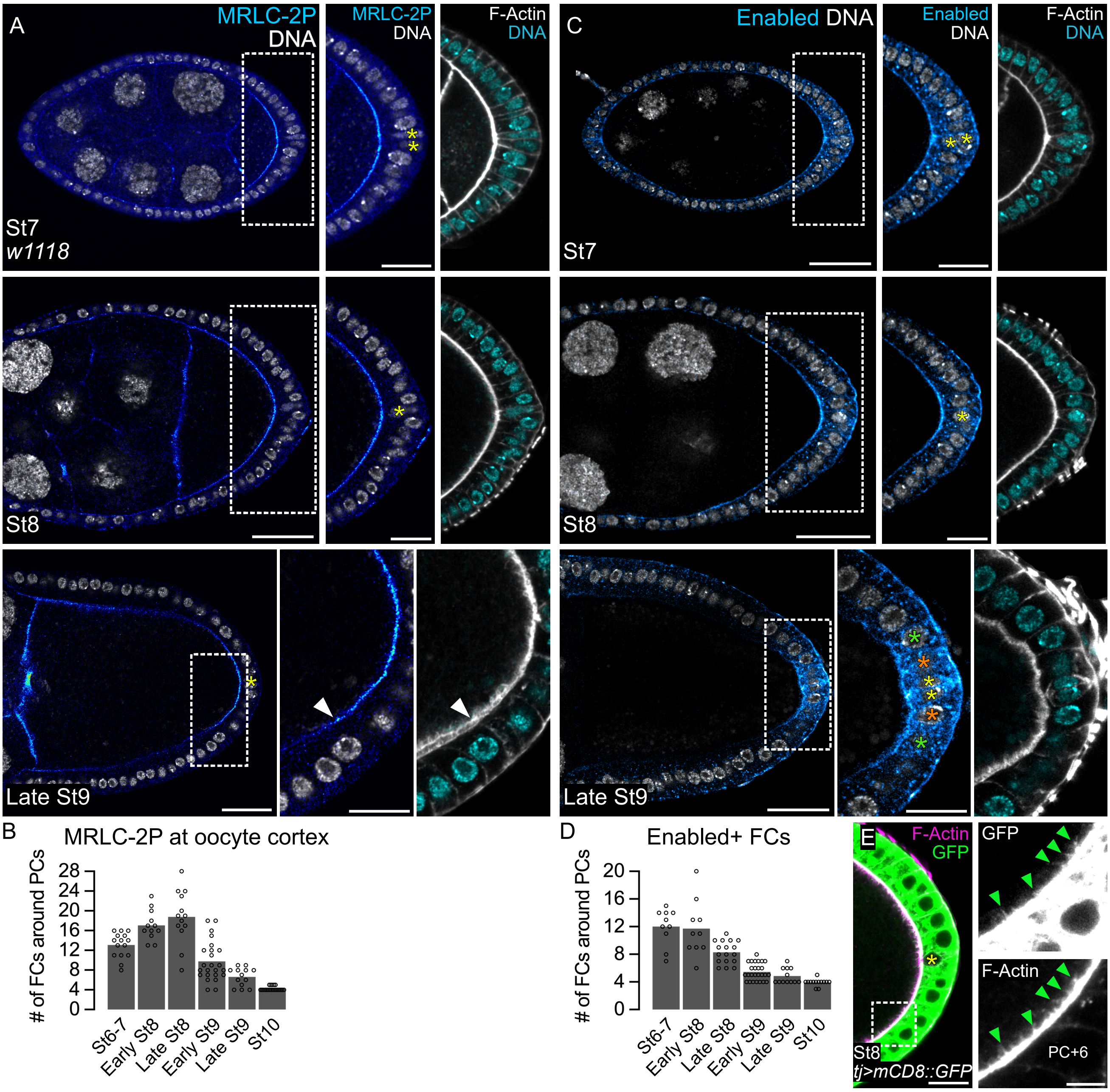

Supplementary Figure 6. MRLC-2P at the oocyte cortex and high Enabled levels in PFC restrict between stages 7 and 10. Posterior is to the right. Yellow, orange and green stars indicate PCs, PC+1 and +2 rows respectively. White dotted rectangles indicate magnifications. **(A,C)** Control follicles (*w[1118]*) stained for F-Actin (grey, phalloidin), DNA (grey or cyan, DAPI) and for MRLC-2P (royal filter) in (A) and for Enabled (cyan hot filter) in (C). In (A), the white arrowhead highlights the boundary where oocyte and PFC membranes separate. **(B,D)** Quantifications in controls follicles of (B) the size of MRLC-2P region as the number of FCs around PCs facing MRLC-2P signal and (D) as the number of Enabled+ FCs around PCs. **(E)** Stage 8 follicle expressing *mCD8::GFP* in FCs under the control of *tj-Gal4*. In merges, GFP is in green and F-Actin in magenta (phalloidin). Green arrowheads point to GFP+/F-Actin+ filopodia.
St: stage. #: number. Scale: 30µm except magnifications 15µm for (A,C) and 5µm for (E). Information about statistics and reproducibility is provided in Supplementary tables 38-40. The data underlying the figure is provided as a Source Data file.

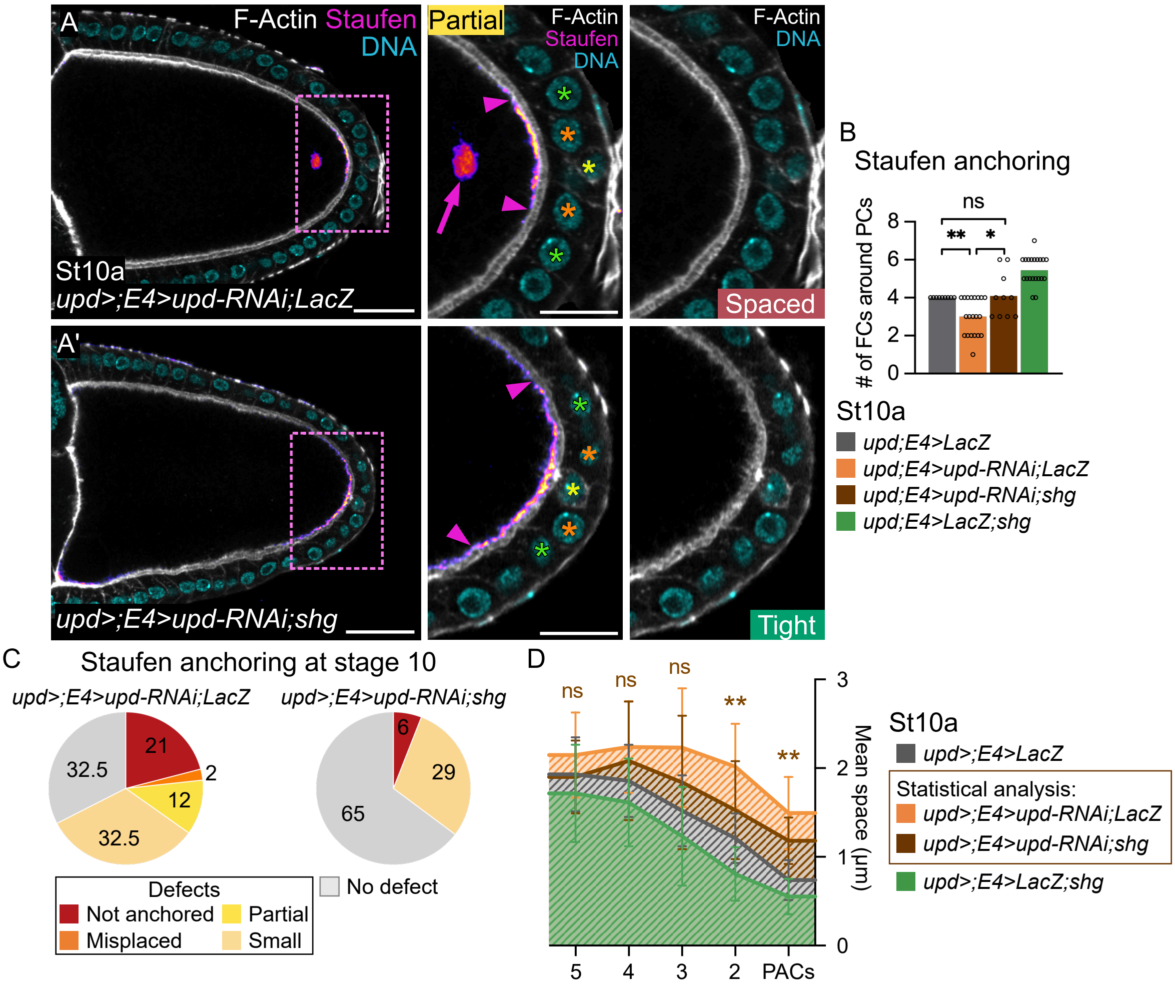

Supplementary Figure 7. E-Cadherin acts downstream of JAK-STAT signaling to maintain PAC-oocyte membranes tight for *oskar* mRNA anchoring in the oocyte. Posterior is to the right. **(A,A')** Stage 10a follicles are stained for F-Actin (grey, phalloidin), DNA (cyan, DAPI) and Staufen (fire filter). PACs (pink dotted rectangles) are magnified. Yellow, orange and green stars indicate PCs, PC+1 and +2 rows respectively. *oskar* mRNA anchoring zone boundary is indicated by pink arrowheads. "Tight" and "Spaced" refer to the perivitelline space. *upd-Gal4* and *E4-Gal4* drive *upd-RNAi* and *LacZ* (A) or *shg* (A') expression in both PCs and PFCs. (A) shows a "Partial" Staufen localization phenotype (smaller anchoring zone and detection in the oocyte cytoplasm, pink arrow) and "large" perivitelline space and (A') shows a rescue of perivitelline spacing ("Tight") and of Staufen localization. **(B,C,D)** Quantification of the anchoring zone size as the number of FCs around PCs facing Staufen signal (B), Staufen posterior localization phenotypes (C) and the perivitelline space (D). In (D), data are presented as mean values +/- SD. In (D), stars indicate statistically significant differences between *upd;E4>updRNAi;LacZ* and *upd;E4>updRNAi;shg* follicles.

St: stage. #: number. Scale: 30µm except magnifications 15µm. Statistical tests: two-sided Mann-Whitney for (B); two-sided unpaired t-test for (D). Information about statistics and reproducibility is provided in Supplementary tables 41-43. The data underlying the figure is provided as a Source Data file.

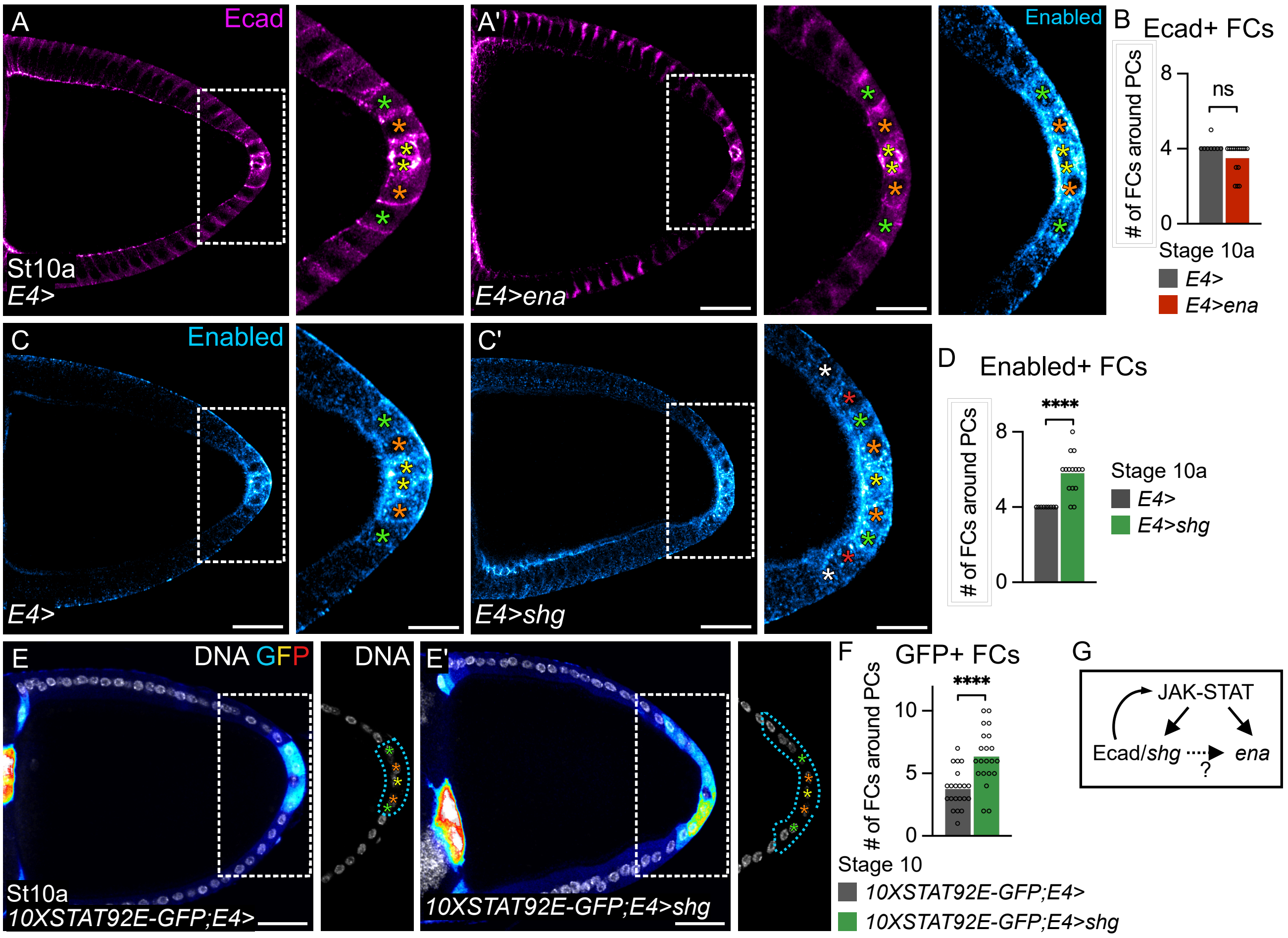

Supplementary Figure 8. *shotgun* is sufficient for ectopic JAK-STAT signaling in PFCs and acts in parallel to *enabled* in PAC differentiation. Posterior is to the right. Yellow, orange, green, red and white stars indicate PCs, PC+1, +2, +3 and +4 rows respectively. PAC region (white dotted rectangles) is magnified. **(A,A',C,C')** Max z-projections of control follicles (*E4/+*) and follicles expressing *ena* or *shg* in PFCs under the control of *E4-Gal4*, stained for E-Cadherin (magenta hot filter) or Enabled (cyan hot filter). **(B,D)** Quantifications of the number of E-Cadherin+ (B) and Enabled+ (D) FCs around PCs in follicles of indicated genotypes. **(E,E')** Sum z-projection of 5 consecutive 1µm confocal sections of control and *E4>shg* follicles expressing *10XSTAT92E-GFP*, stained for DNA (grey, DAPI) and GFP (royal filter). JAK-STAT signaling gradient is outlined in blue. **(F)** Quantifications of the number of GFP+ FCs in the indicated genotypes. **(G)** *ena* and *shg* are parallel targets of the JAK-STAT pathway in PACs. E-Cadherin, as a positive regulator of JAK-STAT signaling, ultimately promotes *ena* expression.
St: stage. #: number. Scale: 30µm except magnifications 15µm. Statistical tests: two-sided Mann-Whitney for (B,D). two-sided unpaired t-test for (F). Information about statistics and reproducibility is provided in Supplementary table 44. The data underlying the figure is provided as a Source Data file.

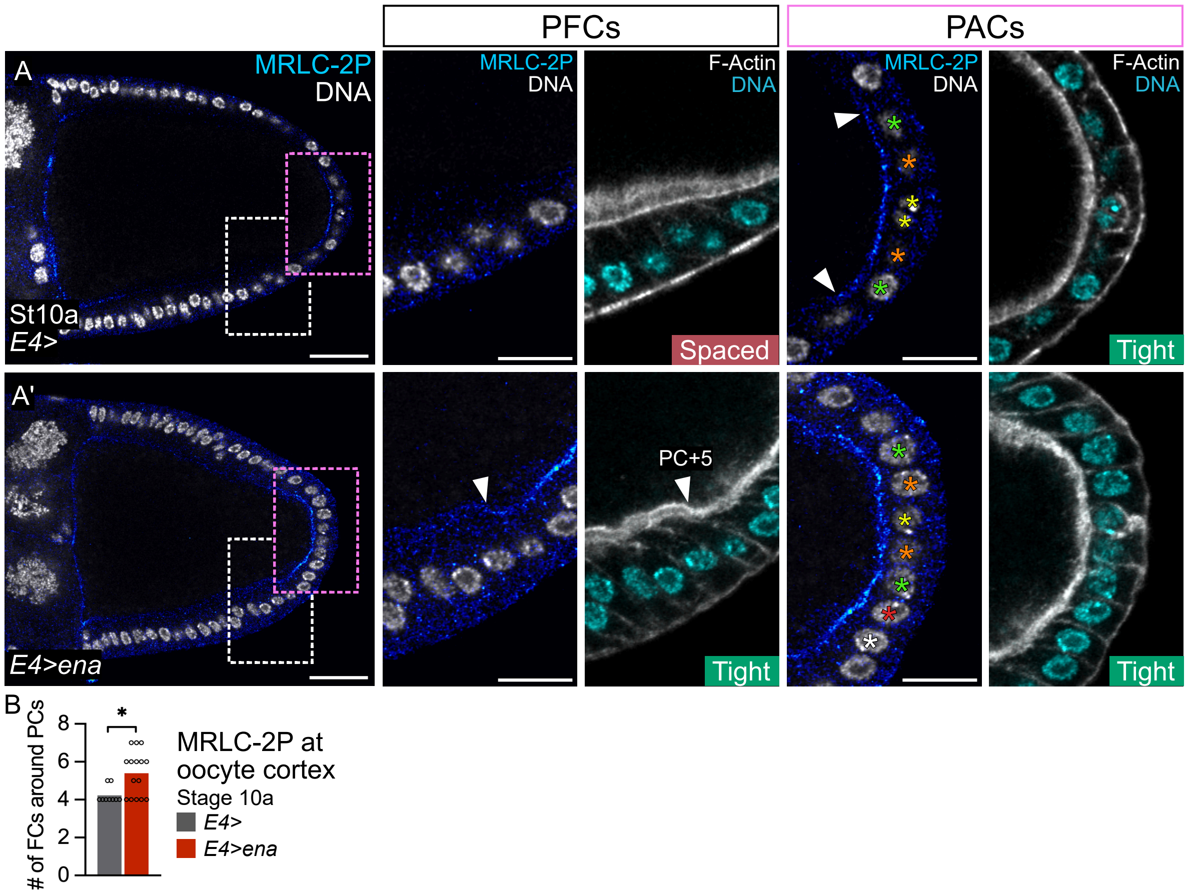

Supplementary Figure 9. *enabled* is sufficient to maintain MRLC-2P at the oocyte posterior cortex. (A,A') Posterior is to the right. Control follicle (*E4/+*) and follicle expressing *ena* in PFCs under the control of *E4-Gal4* stained for F-Actin (grey, phalloidin), DNA (grey or cyan, DAPI) and MRLC-2P (royal filter). Yellow, orange, green, red and white stars indicate PCs, PC+1, +2, +3 and +4 rows respectively. White arrowheads point to the boundary of the region of tight PFC-oocyte contact, that is increased in (A') to PC+5/PC+6. **(B)** Quantification of the size of the MRLC-2P region in number of FCs around PCs facing MRLC-2P signal in control oocytes and when *ena* is expressed ectopically in PFCs.
St: stage. #: number. Scale: 30µm except magnifications 15µm. Statistical tests: two-sided Mann-Whitney. Information about statistics and reproducibility is provided in Supplementary table 45. The data underlying the figure is provided as a Source Data file.
