# Supplementary material for: JAK-STAT-dependent contact between follicle cells and the oocyte controls Drosophila anterior-posterior polarity and germline development
Source: Nat Commun. 2024 Feb 22;15:1627. doi: 10.1038/s41467-024-45963-z (PMC10883949; doi:10.1038/s41467-024-45963-z)
Supplement: Supplementary file 3 — Reporting Summary [file 41467_2024_45963_MOESM3_ESM.pdf]

nature portfolio

Corresponding author(s): Dr Marianne Malartre

Last updated by author(s): Jan 24, 2024

Reporting Summary

Nature Portfolio wishes to improve the reproducibility of the work that we publish. This form provides structure for consistency and transparency in reporting. For further information on Nature Portfolio policies, see our Editorial Policies and the Editorial Policy Checklist.

Statistics

For all statistical analyses, confirm that the following items are present in the figure legend, table legend, main text, or Methods section.

n/a	Confirmed	
☐	☒	The exact sample size (*n*) for each experimental group/condition, given as a discrete number and unit of measurement
☐	☒	A statement on whether measurements were taken from distinct samples or whether the same sample was measured repeatedly
☐	☒	The statistical test(s) used AND whether they are one- or two-sided<br>*Only common tests should be described solely by name; describe more complex techniques in the Methods section.*
☐	☒	A description of all covariates tested
☐	☒	A description of any assumptions or corrections, such as tests of normality and adjustment for multiple comparisons
☐	☒	A full description of the statistical parameters including central tendency (e.g. means) or other basic estimates (e.g. regression coefficient) AND variation (e.g. standard deviation) or associated estimates of uncertainty (e.g. confidence intervals)
☐	☒	For null hypothesis testing, the test statistic (e.g. *F*, *t*, *r*) with confidence intervals, effect sizes, degrees of freedom and *P* value noted<br>*Give P values as exact values whenever suitable.*
☒	☐	For Bayesian analysis, information on the choice of priors and Markov chain Monte Carlo settings
☒	☐	For hierarchical and complex designs, identification of the appropriate level for tests and full reporting of outcomes
☒	☐	Estimates of effect sizes (e.g. Cohen's *d*, Pearson's *r*), indicating how they were calculated

Our web collection on statistics for biologists contains articles on many of the points above.

Software and code

Policy information about availability of computer code

Data collection	Confocal images were acquired with a Leica SP8 inverted microscope driven by Las-X software version 1.4.4.
Data analysis	Quantifications on confocal stacks were performed using FIJI (version 2.14.0/1.54f). All statistical analyses were performed using GraphPad PRISM version 10.1.2 for Mac (GraphPad Software, Boston, Massachusetts USA, www.graphpad.com). Affinity Designer version 2.3 (Serif Europe Ltd, www.affinity.serif.com). All schemes were hand-drawn on Procreate for iPad version 5.3.7 (Savage Interactive Pty. Ltd., www. procreate.com)

For manuscripts utilizing custom algorithms or software that are central to the research but not yet described in published literature, software must be made available to editors and reviewers. We strongly encourage code deposition in a community repository (e.g. GitHub). See the Nature Portfolio guidelines for submitting code & software for further information.

Data

Policy information about availability of data

All manuscripts must include a data availability statement. This statement should provide the following information, where applicable:

- Accession codes, unique identifiers, or web links for publicly available datasets
- A description of any restrictions on data availability
- For clinical datasets or third party data, please ensure that the statement adheres to our policy

All data supporting the findings of this study are available in a Source Data file.

Research involving human participants, their data, or biological material

Policy information about studies with human participants or human data. See also policy information about sex, gender (identity/presentation), and sexual orientation and race, ethnicity and racism.

Reporting on sex and gender	N/A
Reporting on race, ethnicity, or other socially relevant groupings	N/A
Population characteristics	N/A
Recruitment	N/A
Ethics oversight	N/A

Note that full information on the approval of the study protocol must also be provided in the manuscript.

Field-specific reporting

Please select the one below that is the best fit for your research. If you are not sure, read the appropriate sections before making your selection.

☒ Life sciences ☐ Behavioural & social sciences ☐ Ecological, evolutionary & environmental sciences

For a reference copy of the document with all sections, see nature.com/documents/nr-reporting-summary-flat.pdf

Life sciences study design

All studies must disclose on these points even when the disclosure is negative.

Sample size	All biological material (number of cels, follicles or clones) that could be recovered from each genetic condition was used. We ensured that n was always sufficient to give statistically significant results. The sample size is provided in the supplementary data file for each experiment.
Data exclusions	N/A
Replication	All experiments were replicated as indicated in the supplementary data file for each experiment.
Randomization	N/A
Blinding	N/A

Reporting for specific materials, systems and methods

We require information from authors about some types of materials, experimental systems and methods used in many studies. Here, indicate whether each material, system or method listed is relevant to your study. If you are not sure if a list item applies to your research, read the appropriate section before selecting a response.

Materials & experimental systems

n/a	Involved in the study	
☐	☒	Antibodies
☒	☐	Eukaryotic cell lines
☒	☐	Palaeontology and archaeology
☐	☒	Animals and other organisms
☒	☐	Clinical data
☒	☐	Dual use research of concern
☒	☐	Plants

Methods

n/a	Involved in the study	
☒	☐	ChIP-seq
☒	☐	Flow cytometry
☒	☐	MRI-based neuroimaging

Antibodies

Antibodies used	The following primary antibodies were used: rabbit anti-GFP (1:200, Interchim), goat anti-Staufen (1:200, Santa Cruz), rabbit anti-Oskar (1:1000, gift from A. Ephrussi), rat anti-MRLC-2P (1:500, gift from Robert E. Ward), and from DSHB: rat anti-E-Cadherin (1:200), mouse anti-beta-galactosidase (1:200), mouse anti-GFP (1:50), rat anti-vasa (1:200), and mouse anti-Enabled (1:150). Secondary antibodies were all used at 1:200: anti-mouse Cy3 (115-165-003) and anti-rat Alexa 647 (Jackson Laboratories, 112-605-167), anti-rabbit Alexa 488 (A-11008), anti-goat Alexa 488 (A-11055) and 568 (A-11057) and anti-rat Alexa 647 (A-21247) (Thermo Fisher Scientific).
Validation	rabbit anti-GFP (Interchim FP-37151B): tested on transfected cells by manufacturer. anti-Staufen (Santa Cruz): tested on Schneider'DrosophilaLine2wholecell lysate:sc-364794 by manufacturer. anti-Oskar (gift from A. Ephrussi) : original publication DOI:10.1016/0092-8674(91)90137-n. nti-MRLC-2P (gift from Robert E. Ward): original publication DOI:10.1016/j.gep.2010.09.008. anti-E-Cadherin (DSHB): original publication DOI: 10.1006/dbio.1994.1287. anti-beta-galactosidase (DSHB) original publication DOI: 10.1128/mcb.11.12.5848-5859.1991. anti-GFP (DSHB AB_2617419): tested on recombinant GFP expressed in E. coli by manufacturer. anti-vasa (DSHB): original publication DOI: 10.1534/genetics.108.100057. anti-Enabled (DSHB): original publication DOI: 10.1016/s0092-8674(00)80883-1.<br>Secondary antibodies were tested by the manufacturer as follows: anti-mouse Cy3 by immunoelectrophoresis and/or ELISA, anti-rat Alexa 647 by immunoelectrophoresis and/or ELISA, anti-rabbit Alexa 488 by immunohistochemistry and immunocytochemistry, anti-goat Alexa 488 and 568 by flow cytometry,immunohistochemistry and immunocytochemistry and anti-rat Alexa 647 by western blot, immunocytochemistry and immunoprecipitation.

Animals and other research organisms

Policy information about studies involving animals; ARRIVE guidelines recommended for reporting animal research, and Sex and Gender in Research

Laboratory animals	Drosophila melanogaster. All strains were used for dissection of ovaries between 3 and 5 days after eclosion. The following stocks were used: 10XSTAT92E-GFP (DOI: 10.1016/j.modgep.2006.08.003), shg-LacZ (gift from JP. Vincent), Khc::LacZ (DOI: 10.1016/s0960-9822(00)00068-3), UAS-hopTUM (DOI: 10.1002/j.1460-2075.1995.tb07285.x), grk2E12, Df(2L)ED629 (Gifts from V. Mirouse), traffic-jam Gal4 (DGRC, Kyoto), fru-Gal4 (DOI: 10.1242/dev.079046), E4-Gal4 (DOI: 10.1242/dev.124.19.3871), upd-Gal4 (doi:10.1038/cdd.2010.141), UAS GBP-APEX2 (DOI: 10.3389/fcell.2021.719582) and oskar::GFP (gift from A. Ephrussi). RNAi strains UAS-upd-RNAi (P{GD1158}v3282), UAS-EGFR-RNAi (P{KK100051}VIE-260B) are from VDRC. w1118, UAS-mCD8::GFP, hsFLP;tub<CD2<Gal4,UAS-GFP, nanos-Gal4VP16, UAS-ena::mcherry, UAS-shg.R, UAS-shg-RNAi (P{TRiP.GL00646}attP40), UAS-EGFR-RNAi (P{TRiP.JF01084}attP2), pnt-LacZ (P{PZ}pnt[07825]) and UAS-LacZ (P{UAS-lacZ.NZ}J312) are from BDSC.
Wild animals	This study did not involve wild animals.
Reporting on sex	These findings only apply to female as they were conducted using Drosophila ovary as a model system.
Field-collected samples	This study did not involve samples collected from the field.
Ethics oversight	No ethical approval or guidance was required as it is not necessary for studies on Drosophila.

Note that full information on the approval of the study protocol must also be provided in the manuscript.

Plants

Seed stocks	N/A
Novel plant genotypes	N/A
Authentication	N/A
